# *In vitro* activity of cefiderocol against nosocomial *Acinetobacter baumannii*

**DOI:** 10.1128/spectrum.00844-25

**Published:** 2025-10-08

**Authors:** Barbara Ymaña, Rocío Egoávil-Espejo, Rosario Huerto-Huánuco, Rosario Oporto-Llerena, Carla A. Alonso, Angie K. Castillo, Luciano A. Palomino-Kobayashi, Carmen Valera-Krumdieck, Gabriela Soza, Tamin Ortiz-Gomez, Patricia Gonzales, María López, Gina Salvador-Luján, Beatriz Rojo-Bezares, Martin Casapia, Paula Toledano, Joseph Pinto, Maria Ramos Chirinos, Yolanda Sáenz, Maria J. Pons, Joaquim Ruiz

**Affiliations:** 1 Grupo de Investigación en Dinámicas y Epidemiología de la Resistencia a Antimicrobianos - “One Health”, Universidad Científica del Sur, Lima, Peru; 2 Servicio de Análisis Clínicos, Laboratorio de Microbiología, Hospital Universitario San Pedro, Logroño, Spain.; 3 Servicio de Patología Clínica área Microbiología, Hospital María Auxiliadora, Lima, Peru; 4 Instituto Nacional Materno Perinatal 281312 , Lima, Peru; 5 Servicio de Microbiología y Biología Molecular, Laboratorios AUNA, Lima, Peru; 6 Servicio de Microbiología, Hospital María Auxiliadora, Lima, Peru; 7 Área de Microbiología Molecular, Centro de Investigación Biomédica de La Rioja 541787, Logroño, Spain; 8 Laboratorio de Microbiología, Hospital Militar Central, Lima, Peru; 9 Facultad de Ciencias Biológicas, Universidad Nacional, Mayor de San Marcos, Lima, Peru; 10 Hospital Regional de Loreto, Iquitos, Peru; 11 Facultad de Medicina Humana, Universidad Nacional de la Amazonia Peruana, Iquitos, Peru; 12 Centro de Investigación Básica y Traslacional Auna Ideas, Lima, Peru; UCI Health, Orange, California, USA

**Keywords:** cefiderocol, beta-lactamases, antibiotic resistance, middle-income countries, carbapenemases, OXA-24

## Abstract

The emergence and spread of third-/fourth-generation cephalosporin and/or carbapenem-resistant *Acinetobacter baumannii* have become a significant global public health concern, making new treatment alternatives necessary. Thus, the present study aimed to assess *in vitro* cefiderocol activity against clinical isolates of *A. baumannii* and analyze their relationship with extended-spectrum β-lactamases (ESBLs) and carbapenemases. Ninety-five *A. baumannii* clinical isolates were included in the study. Susceptibility to 12 antimicrobial agents was established by automated methods and/or disk diffusion, while that of colistin was determined following microdilution and that of cefiderocol by microdilution using iron-depleted broth. The presence of *bla*_CTX-M_, *bla*_PER_, *bla*_VEB_, *bla*_GES_, *bla*_VIM_, *bla*_IMP_, *bla*_IMI_, *bla*_KPC_, *bla*_NDM_, *bla*_OXA-23G_, *bla*_OXA-24G_, *bla*_OXA-48G_, and *bla*_OXA-58G_ was established by PCR. The results showed extremely high levels of resistance (>80%) to all the tested antibacterial agents except colistin (11.6%) and cefiderocol (Clinical and Laboratory Standards Institute [CLSI]: 0%; US Food and Drug Administration [FDA]: 1.1%). Following FDA criteria, 22.1% of isolates were intermediate to cefiderocol, with 68.4% of isolates surpassing the European Committee on Antimicrobial Susceptibility Testing epidemiological cut off. Seven colistin-resistant isolates were only susceptible to cefiderocol following CLSI breakpoints, four of them qualifying as cefiderocol-intermediate following FDA breakpoints. No association between the presence of ESBLs or carbapenemases and cefiderocol minimum inhibitory concentration levels was observed. The present results show the potential utility of cefiderocol in the treatment of *A. baumannii* infections, highlighting the need for judicious use and continuous surveillance to prevent the emergence of cefiderocol-resistant *A. baumannii* clones.

**IMPORTANCE:**

Antibiotic resistance is a silent pandemic challenging the treatment of infectious diseases worldwide, but also other medical practices, as, for instance, organ transplantation procedures. In Peru, current levels of antimicrobial resistance are worrisome. In this scenario, we have determined the *in vitro* activity of cefiderocol against a series of *Acinetobacter baumannii* exhibiting high levels of resistance to commonly used antibiotics. This activity is independent of the presence of the most common extended-spectrum beta-lactamases or carbapenemases. Obtained results showed the potential of cefiderocol to become an alternative for the treatment of this type of microorganism, but the high number of isolates bordering the considered breakpoint, despite the lack of use of cefiderocol in the country, also shows the need for a prudent use of this antibiotic to maximize its utility while minimizing the selection of resistant isolates.

## INTRODUCTION

Antimicrobial resistance is a growing phenomenon affecting microorganisms of all environments (1–4), but it is especially worrisome in clinical settings, where the lives of the most vulnerable patients may be at risk (5). This problem not only jeopardizes the effectiveness of antibiotic treatments when needed, but it also has a serious impact on other human health aspects, such as surgical prophylaxis or the use of antibiotics in at-risk populations, such as post-transplant patients (6, 7).

In fact, antimicrobial resistance is considered one of the leading public health threats of the 21st century and also severely impacts both direct and indirect economic costs (8, 9), qualifying among the World Health Organization (WHO) urgent health challenges for the decade (https://www.who.int/news-room/photo-story/detail/urgent-health-challenges-for-the-next-decade). In this sense, the most recent data have shown that antibiotic resistance was the cause of 1.27 million deaths in 2019 and was involved in an additional 4.95 million deaths (10). Although all microorganisms and environments may be affected, a series of microorganisms are of special concern, being collectively known under the acronym ESKAPE (*Enterococcus faecium, Staphylococcus aureus, Klebsiella pneumoniae, Acinetobacter baumannii, Pseudomonas aeruginosa,* and *Enterobacter cloacae*) or E-ESKAPE, ESKAPE-Ec when also considering *Escherichia coli*.

As indicated above, *A. baumannii* is a member of the ESKAPE group, which is often isolated as a cause of infection in patients attending intensive care units (11, 12). Carbapenems have been considered the treatment of choice for this microorganism, but the increase in the current rates of carbapenem resistance alerts about the need for alternative treatment strategies (13). Indeed, carbapenem-resistant *A. baumannii* (CR*Ab*) is included among the WHO critical priority microorganisms due to the lack of alternative treatments (https://www.who.int/news/item/17-05-2024-who-updates-list-of-drug-resistant-bacteria-most-threatening-to-human-health), which are often limited to colistin or a combination of antibacterial agents (13, 14). Of note, *A. baumannii* exhibiting colistin resistance has been isolated in different areas, opening the door to the isolation of pan-drug-resistant isolates (12, 15, 16).

In this scenario, new alternatives able to fight infections by CR*Ab* are an emerging need worldwide. Cefiderocol is a recently developed siderophore cephalosporin, which enters the bacterial cell through classical cephalosporin routes (17), as well as via iron transporters thanks to a C-3 side chain, which has a chlorocatechol group at the end of the C-3 side chain that confers the above-indicated siderophore ability (17, 18). This increased intake, together with a high degree of stability versus the activity of a great variety of β-lactamases, also related to the above-mentioned chlorocatechol group (17), results in promising activity against a variety of Gram-negative microorganisms, including *A. baumannii* (19, 20).

Most of the studies published on cefiderocol activity have been conducted in high-income countries (19–21), making studies of microorganisms of clinical interest from other areas, such as Peru, necessary. Peru is a middle-income country in which current data about CR*Ab* have shown their presence in a large series of health centers, with resistance rates to other antimicrobial agents commonly being higher than 50%. (15, 16, 22), and with emerging descriptions of colistin-resistant isolates (15, 16).

Thus, this study aimed to determine the *in vitro* activity of cefiderocol against third-/fourth-generation cephalosporins and/or CR*Ab* isolated in different areas of Peru.

## RESULTS

Eighty-nine isolates were confirmed as *A. baumannii* by matrix-assisted laser desorption/ionization time-of-flight (MALDI-TOF). The remaining six were classified as *A. baumannii* following the amplification of *bla*_OXA-51_ and/or the amplification and sequencing of 16S rRNA.

Overall, high levels of antimicrobial resistance were observed. Thus, the *A. baumannii* isolates presented non-susceptibility levels higher than 90% to almost all classical antibacterial agents, except for ampicillin plus sulbactam (83.2%), ceftazidime plus avibactam (87.4%), gentamicin (89.5%), and colistin (11.6%) (Table 1). Not taking into account cefiderocol, 7 out of 11 (63.6%) colistin-resistant isolates showed non-susceptibility to all antibacterial agents included in the study and were thereby classified as potential pan-drug-resistant isolates. Three of these isolates presented a minimum inhibitory concentration (MIC) of cefiderocol of 1 µg/mL, qualifying as susceptible both by Clinical and Laboratory Standards Institute (CLSI) and U.S. Food and Drug Administration (FDA) breakpoints. The remaining four presented a MIC of 2 µg/mL and were therefore classified as intermediate by FDA breakpoints.

**TABLE 1:** Susceptibility to antibacterial agents^*a*^

AA	S (No, %)	I (No, %)	R (No, %)	NS (No, %)
SAM	16 (16.8)	16 (16.8)	63 (66.3)	79 (83.2)
TZP	3 (3.2)	4 (4.2)	88 (92.6)	92 (96.8)
ATM	1 (1.1)	7 (7.4)	87 (91.6)	94 (98.9)
CTX	0 (0.0)	3 (3.2)	92 (96.8)	95 (100.0)
CAZ	4 (4.2)	3 (3.2)	88 (92.6)	91 (95.8)
FEP	7 (7.4)	8 (8.4)	80 (84.2)	88 (92.6)
CZA	12 (12.6)	–	83 (87.4)	83 (87.4)
IPM	4 (4.2)	0 (0.0)	91 (95.8)	91 (95.8)
MEM	3 (3.2)	1 (1.1)	91 (95.8)	92 (96.8)
CIP	6 (6.3)	0 (0.0)	89 (93.7)	89 (93.7)
GEN	10 (10.5)	7 (7.4)	78 (81.1)	85 (89.5)
AMK	9 (9.5)	5 (5.3)	81 (83.5)	86 (90.5)
CST	84 (88.4)	–	11 (11.6)	11 (11.6)
FDC (FDA)	73 (76.8)	21 (22.1)	1 (1.1)	22 (23.2)
FDC (CLSI)	95 (100.0)	0 (0.0)	0 (0.0)	0 (0.0)

^*a*^ AA, antimicrobial agent; S, susceptible; I, intermediate; R, resistant; NS, non-susceptible SAM, ampicillin plus sulbactam; TZP, piperacillin plus tazobactam; ATM, aztreonam; CTX, cefotaxime; CAZ, ceftazidime; FEP, cefepime; CZA, ceftazidime plus avibactam; IPM, imipenem; MEM, meropenem; CIP, ciprofloxacin; GEN, gentamicin; AMK, amikacin; CST, colistin; FDC, cefiderocol; FDA, U.S. Food and Drug Administration; CLSI, Clinical and Laboratory Standards Institute breakpoint. The hyphen symbolizes the non-established intermediate category.

**Fig 1:**
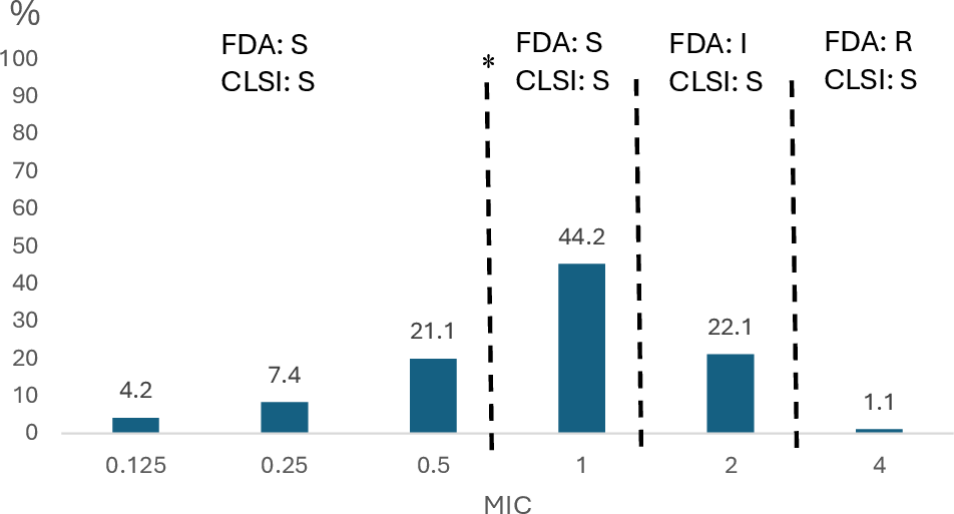
MIC (µg/ml) distribution. The asterisk marks the alert of the European Committee on Antimicrobial Susceptibility Testing regarding risk isolates (23).

Regarding cefiderocol, the MICs ranged from 0.125 µg/mL to 4 µg/mL, with a MIC_50_ and MIC_90_ of 1 µg/mL and 2 µg/mL, respectively. Only one isolate presented a MIC = 4 µg/mL being classified as susceptible according to CLSI criteria, but resistant by FDA criteria. Furthermore, following the FDA breakpoints, additional 21 (22.1%) isolates were classified as intermediate, resulting in 22 (23.2%) non-susceptible to cefiderocol based on FDA criteria (Table 2; Fig. 1).

**TABLE 2:** Distribution of the MICs of cefiderocol

					MIC cefiderocol (µg/ml)					
	No	MIC_50_	MIC_90_	Mode	0.125	0.25	0.5	1	2	4
*A. baumannii*	95	1	2	2	4	7	19	43	21	1
CLSI^*a*^					S	S	S	S	S	S
FDA^*b*^					S	S	S	S	I	R

^*a*^ CLSI, Clinical and Laboratory Standards Institute.

^*b*^ FDA, U.S. Food and Drug Administration.

On bacterial population terms, following the European Committee on Antimicrobial Susceptibility Testing (EUCAST), 65 (68.4%) isolates were over the epidemiological cut-off (ECOFF) of 0.5 µg/mL.

Regarding extended-spectrum β-lactamases (ESBLs), no *bla*_CTX-M_ was found, with three isolates presenting *bla*_VEB_, one *bla*_PER_, and one *bla*_GES_ (while the latter was classified here as ESBL, in the absence of a full gene amplification and sequencing, it cannot be definitively determined as an ESBL or carbapenemase). Interestingly, all ESBLs were present concomitantly with a carbapenemase.

The most common carbapenemase genes belonged to the *bla*_OXA-24_ group, which was present alone in 71 (74.7%) isolates, and concomitantly with another carbapenemase or an ESBL in other 7 (7.4%) isolates: *bla*_VEB_ in 3 cases and with *bla*_PER_, *bla*_OXA-23G_, *bla*_OXA-48G_, and *bla*_NDM_ in one isolate each. The *bla*_OXA-23_ gene was found alone in 8 (8.4%) isolates, and together with a *bla*_GES_ in one isolate. The *bla*_NDM_ gene was also present alone in another isolate. Seven (7.4%) isolates did not present any ESBL or carbapenemase sought (Table 3).

**TABLE 3:** Association of ESBLs and carbapenemases with final MIC levels to cefiderocol

			Cefiderocol MIC					
Carbapenemases	ESBLs^*a*^	No	0.125	0.25	0.5	1	2	4
---^*b*^	---	7			2	5		
NDM	---	1					1	
OXA-23G	---	8	1			5	2	
OXA-24G	---	71	3	5	16	31	16	
OXA-23G	GES^*c*^	1		1				
OXA-24G	VEB	3				1	1	1
OXA-24G	PER	1			1			
OXA-24G + OXA-23G	---	1			1			
OXA-24G + OXA-48G	---	1		1				
OXA-24G + NDM	---	1					1	

^*a*^ ESBLs, extended-spectrum β-lactamases.

^*b*^ The lack of carbapenemases and/or ESBLs is noted by hyphens.

^*c*^ Although classified as ESBL, in the absence of a full-gene amplification and sequencing, it cannot be definitively determined as an ESBL or carbapenemase.

No association was observed between the presence of ESBLs or carbapenemases and the final cefiderocol MIC levels, with the isolate with the highest MIC of cefiderocol (4 µg/mL), only being susceptible to ampicillin plus sulbactam and colistin and presenting *bla*_VEB_ and *bla*_OXA-24G_.

## DISCUSSION

Resistance to antibacterial agents is an increasingly worrisome problem that seriously challenges current medical practice. While this phenomenon affects all countries and environments, it is of special concern in low- and medium-income countries. These countries usually present economic restrictions, which often lead to limited treatment alternatives, and/or restrictions or difficulty in achieving access to health facilities, safe water, or sanitation, in addition to social factors including over-the-counter access to antibacterial agents and lack of health education among most of the population (24).

In Peru, most reports describe a dreadful panorama, with prevalence of self-medication of antibiotics around 50%, extremely high levels of resistance to a variety of antibacterial agents by pathogens isolated in health facilities (11, 15, 25), and the isolation of multidrug-resistant or extensively drug-resistant microorganisms outside clinical settings. The present data confirm these high levels of resistance to antimicrobial agents in CR*Ab*, with colistin as the only alternative showing resistance levels less than 80%. Nevertheless, colistin should be used with caution because of its toxicity (26). Furthermore, resistance to this agent is emerging in the area (15, 16), with this issue being also confirmed in the present study. Although data are scarce, in Peru, fatality rates of 42.9% have been described in patients with bloodstream infections (16) and of 27.6% in patients with extensively drug-resistant *A. baumannii* infections treated with colistin (26). These findings indicate the urgent need to introduce new treatment alternatives in the area.

Cefiderocol is a new antibacterial agent, a siderophore-cephalosporin, which has shown good activity levels against relevant pathogens, including *A. baumannii* (19, 20). Although cefiderocol has yet to be introduced in clinical practice in Peru, it may be a potential alternative to treat pathogens, such as CR*Ab*, and therefore evaluation of its *in vitro* activity is important.

The present results show that cefiderocol is, by and large, the most active antibacterial agent tested versus the current collection of *A. baumannii* analyzed (almost all CR*Ab*), regardless of the presence of ESBLs or carbapenemases, as shown by other studies (27, 28). No isolate was found to be resistant according to CLSI breakpoints, but one was resistant, and 21 showed intermediate resistance following FDA criteria. This difference between the two criteria is related to the high number of isolates with a MIC = 2 µg/mL. With respect to colistin-resistant isolates, the MIC of cefiderocol was 1–2 µg/mL, being a relatively high MIC according to EUCAST ECOFF (29). Of note, EUCAST, in the line of FDA breakpoints, suggests that cefiderocol MICs of 1–2 µg/mL may lead to an impaired clinical response, with 64 isolates (67.4%) presenting MIC values ≥1 µg/mL (25). The above-mentioned data highlight a serious question about the lack of standardization of susceptibility breakpoints, which might impact the correct patient management.

Studies developed in other areas show levels of cefiderocol resistance higher than those observed in the present study. Thus, a recent study analyzing 402 carbapenem-resistant *Acinetobacter calcoaceticus-baumannii* complex from the United States showed rates of resistance of 7% and 20.6%, as for CLSI and FDA, respectively, with additional series of intermediate isolates (30). Similarly, a study developed in Vietnam showed c. 15% of resistance (as for CLSI breakpoints) among *A. baumannii* colonizing ICU patients (31). Differences in the origin of samples and/or methodologies to determine susceptibility levels as well as the levels of cefiderocol use in clinical practice may underline the differences in the levels of cefiderocol resistance.

In the present series, the *bla*_OXA-24G_ genes were the most common carbapenemases among the isolates analyzed, with *bla*_OXA-23G_ being the second most common. The presence of other carbapenemases was testimonial. This agrees with studies in the area in which the presence of members of these OXA groups has been identified as the most common carbapenemases in CR*Ab*. Thus, different authors have described a scenario in which members of the *bla*_OXA-24G_ (e.g., *bla*_OXA-72_) were the most common carbapenemases amongst CR*Ab*, followed by *bla*_OXA-23G_ (22, 32, 33).

While the role of *bla*_OXA-24G_ and *bla*_OXA-23G_ in the development of cefiderocol resistance seems null (28), it has been proposed that specific β-lactamases, such as those belonging to the *bla*_PER_ and *bla*_NDM_ families, might play a role in the development of resistance to cefiderocol (34, 35). In the present study, the low number of isolates possessing these β-lactamases does not allow conclusions to be obtained, with the only isolate possessing *bla*_PER_ showing a MIC of 0.5 µg/mL, and the two isolates possessing *bla*_NDM_ presenting MICs of 2 µg/mL, therefore classified as intermediate following FDA criteria, and being over the EUCAST ECOFF. Nevertheless, these MIC values are within the range of those obtained by Poirel et al. when cloned several PER and NDM encoding genes in *A. baumannii* CIP70.10, showing that, while contributing to the final cefiderocol MIC levels, these alone are not enough to clearly surpass the established breakpoints (34).

The analysis of cefiderocol susceptibility showed a scenario in which 68.4% of isolates were over EUCAST ECOFF (29). This finding strongly suggests that, apart from the scarce presence of NDM or PER, the presence of mechanisms able to slightly increase the MIC to cefiderocol should be considered. The present data highlight not only cefiderocol as a potential alternative to current treatments for *A. baumannii* infections but also the need to use this agent with caution.

While approved in countries such as the United States, country members of the European Union, United Kingdom, or Japan, and with application under study in others, such as Australia (https://www.shionogi.com/global/en/news/2025/04/20250402.html; https://www.shionogi.com/global/en/news/2022/06/20220616.html), cefiderocol is accessible under early access programs (compassionate use) in a long series of countries, mostly qualifying as low- or middle-income countries (https://www.shionogi.com/us/en/innovation/expanded-access-policy.html; https://www.inceptua.com/inceptua-group-expands-early-access-program-to-latin-america/). In low- and middle-income countries, stable access to modern antimicrobial agents has a series of challenging questions, with the antibiotic cost as a severe limiting factor (36). Regarding cefiderocol, in June 2022, Shionogi, the Global Antibiotic Research and Development Partnership, and The Clinton Health Access Initiative signed an agreement to facilitate the access to cefiderocol in 135 countries (https://www.shionogi.com/global/en/news/2022/06/e220615.html).

The correct identification of *A. baumannii* is challenging, because several closely related species may be indistinguishable or easily mistakenly identified. MALDI-TOF is a reliable methodology, but the presence of bacterial misidentification has also been reported (37), and the continuous description of new species may result in different bacterial identification as for version of database (38). The non-identification of the specific β-lactamases is one of the limitations of the study, but the results showed good parameters of cefiderocol activity, irrespective of the presence or absence of the most common *A. baumannii* β-lactamases. While not a limitation, the inclusion criteria should be taken into account, and therefore the present levels of antimicrobial resistance are those of third-/fourth-generation cephalosporin-resistant *A. baumannii* and/or CR*Ab*. In any case, the levels of resistance to these agents in Peru among *A. baumannii* isolates are extremely high (16, 39), surpassing 80% according to some reports (15).

At present, cefiderocol remains to be introduced in clinical practice in the area. *In vitro* activity levels showed that cefiderocol might play a role in the treatment of extensively drug-resistant CR*Ab*, but MIC values highlight the need for judicious use and continuous surveillance to avoid or minimize the risk of the development of resistance.

## MATERIALS AND METHODS

### Microorganisms

Ninety-five non-duplicate third-/fourth-generation cephalosporins and/or CR*Ab* were isolated from different clinical samples in different health centers in Peru between 2020 and 2022 (Table 4). All isolates were identified by VITEK-2 as belonging to the *Acinetobacter calcoaceticus-baumannii* complex and were sent to the Hospital Universitario San Pedro (Logroño, Spain) to be confirmed at the species level by MALDI-TOF using the MBT Compass Library V11.0.0.0 (July 2021). Isolates with no conclusive MALDI-TOF results were confirmed by the amplification of an internal 353 bp fragment of *bla*_OXA-51_ and/or amplification and sequencing of 16S rRNA (40, 41).

**TABLE 4:** Origin of the samples^*a*^

Health center	No	City	Location	Region
A	53	Lima	Metropolitan Lima	Coast
B	14	Lima	Metropolitan Lima	Coast
C	12	Lima	Metropolitan Lima	Coast
D	8	Lima	Metropolitan Lima	Coast
E	3	Iquitos	Loreto (Northern Peru)	Jungle
F	2	Lima	Metropolitan Lima	Coast
G	2	Piura	Piura (Northern Peru)	Coast
ND	1	Lima	Metropolitan Lima	Coast

^*a*^ ND, not determined.

### Susceptibility to antimicrobial agents

The susceptibility levels to ampicillin plus sulbactam, piperacillin plus tazobactam, aztreonam, cefotaxime, ceftazidime, cefepime, ceftazidime plus avibactam, imipenem, meropenem, ciprofloxacin, gentamicin, and amikacin were established using disk diffusion. Susceptibility to colistin was established according to CLSI guidelines (42). Susceptibility to cefiderocol was determined by microdilution in iron-depleted broth (Remel, Lenexa, USA) as previously described (42, 43). Briefly, a preliminary MIC was established using a range of cefiderocol concentrations containing from 0.06 µg/mL to 2 µg/mL, in the wells of 96-microwell plates. Isolates growing on wells containing 2 µg/mL cefiderocol were tested again in 96-well plates containing up to 64 µg/mL of cefiderocol. A growth control (tested bacteria cultured in media without cefiderocol) and a blank (non-inoculated media without cefiderocol) were used as controls in all determinations. Plates were read at 600 nm in an enzyme-linked immunosorbent assay reader (SYNERGY LX, Biotek, Santa Clara, USA). Bacterial growth was considered when the optical density (OD) was >0.100 (approximately 2.5 times higher than absorbance values of blank wells) (43). Isolates with absorbance values ranging from 0.09 to 0.110 were classified as uncertain and tested again (43).

The susceptibility data were interpreted according to CLSI (42). In the absence of a specific breakpoint, data of ceftazidime-avibactam were interpreted based on *P. aeruginosa* breakpoints. In addition, cefiderocol susceptibility was also interpreted as per the FDA guidelines (44).

As an internal control, susceptibility levels were determined up to three times in a series of randomly selected isolates, as well as in those growing on ≥2 µg/mL of cefiderocol. Susceptibility differences of one dilution were considered as an inherent methodological error. The *E. coli* ATCC 25922 was used as the quality control in all experiments.

In the text, the term non-susceptible refers to the sum of intermediate and resistant isolates.

In addition, the ecological distribution of MICs was also analyzed following EUCAST guidelines (29).

### ESBL and carbapenemase detection

The presence of the ESBL families CTX-M, PER and VEB, GES (ESBLs or carbapenemases), and carbapenemases type VIM, IMP, IMI, KPC, NDM, OXA-23, OXA-24, OXA-48, and OXA-58 was determined by PCR as previously described (Table 5) (45–48). In the text, the OXA-encoding genes sought are indicated with a final “G” or with the word “group” to avoid misinterpretation, because the primers used allow the amplification of a series of related genes.

**TABLE 5:** Primers used in this study to detect ESBLs and carbapenemases

	Primer sequence				
Gene	Forward (5' → 3')	Reverse (5' → 3')	Size^*a*^	Ann^*b*^	Ref^*c*^
*bla*_CTX-M-like_	CGATGTGCAGTACCAGTAA	TTAGTGACCAGAATCAGCGG	585	60	(48)
*bla*_GES_	CTGGCAGGGATCGCTCACTC	TTCCGATCAGCCACCTCTCA	600	57	(45)
*bla*_PER_	AGTGTGGGGGCCTGACGAT	GCAACCTGCGCAATRATAGCTT	725	57	(45)
*bla*_VEB_	CGACTTCCATTTCCCGATGC	TGTTGGGGTTGCCCAATTTT	376	57	(45)
*bla*_KPC_	TCGCCGTCTAGTTCTGCTGTCTTG	ACAGCTCCGCCACCGTCAT	353	57	(45)
*bla*_NDM_	ACTTGGCCTTGCTGTCCTT	CATTAGCCGCTGCATTGAT	603	57	(45)
*bla*_IMI_	CTACGCTTTAGACACTGGC	AGGTTTCCTTTTCACGCTCA	482	57	(47)
*bla*_VIM_	TGTCCGTGATGGTGATGAGT	ATTCAGCCAGATCGGCATC	437	57	(45)
*bla*_IMP_	ACAYGGYTTRGTDGTKCTTG	GGTTTAAYAAARCAACCACC	387	57	(45)
*bla*_OXA-23-like_	TACAAGGGATTCGGCATCG	TAATGGCCTGTTCCCATGTG	570	52	(46)
*bla*_OXA-24-like_	AAAATCTGGGTACGCAAACG	ACATTATCCGCTGGAACAGG	271	52	(46)
*bla*_OXA-48-like_	ATGCGTGTATTAGCCTTATCG	CATCCTTAACCACGCCCAAATC	265	57	(45)
*bla*_OXA-58-like_	TCGACACACCTTGGTCTGAA	AACTTCCAACTTTGCCATGC	477	52	(46)

^*a*^ Size in base pairs (bp).

^*b*^ Annealing temperature (°C).

^*c*^ References.
